# Concomitant Bifocal Urothelial Carcinoma and Breast Tumor: Second Primary Cancer or Metastatic Spread to the Breast?

**DOI:** 10.1155/2014/917581

**Published:** 2014-08-04

**Authors:** Clément Dumont, Hélène Gauthier, Jérôme Vérine, Jacqueline Lehmann-Che, Patricia de Cremoux, Damien Pouessel, Stéphane Culine

**Affiliations:** ^1^Department of Medical Oncology, AP-HP, Saint-Louis Hospital, University Paris Diderot, 75010 Paris, France; ^2^Department of Pathology, AP-HP, Saint-Louis Hospital, University Paris Diderot, 75010 Paris, France; ^3^Molecular Oncology Unit, Department of Biochemistry, AP-HP, Saint-Louis Hospital, University Paris Diderot, 75010 Paris, France

## Abstract

Metastases to the mammary gland are an uncommon event in the natural history of most malignant tumors. We report the case of a 60-year-old woman who presented initially with bifocal urothelial carcinoma with a single breast tumor, raising the issue of a primary cancer or a metastatic spread to the breast. The diagnosis of breast metastasis was aided by identity of pathology, immunochemistry, and molecular biology findings between the primary tumor and the breast lesion, among which are the p.K120M mutation, a very rare TP53 mutation, and HER2 overexpression with underlying polysomy of chromosome 17.

## 1. Introduction

Metastases to the mammary gland are an uncommon event in the natural history of most malignant tumors, with incidence ranging from 0.12% to 4.92% in various tumor types [1]. Only four cases of breast metastasis from urothelial carcinoma (UC) have been reported so far in the literature [2–5]. Here we report the case of a 60-year-old woman who presented initially with bifocal UC with a single breast tumor, raising the issue of a primary cancer or a metastatic spread to the breast.

## 2. Case Report

**Figure 1:**
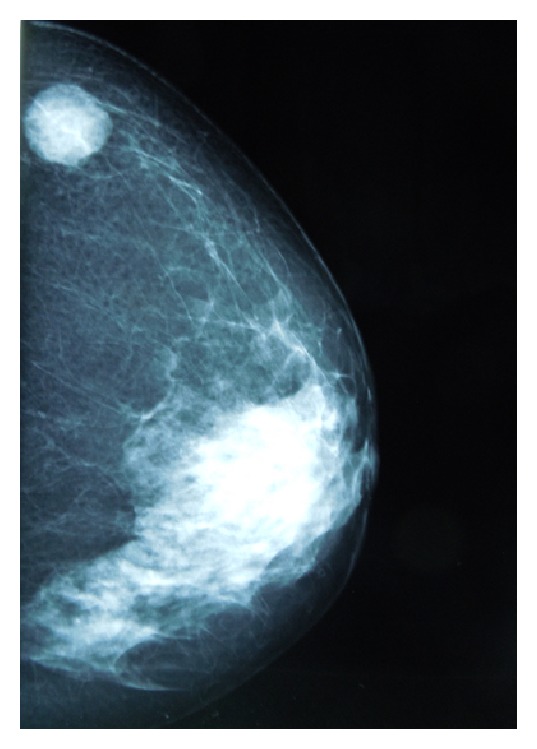
Mammography showing breast tumor.

**Figure 2:**
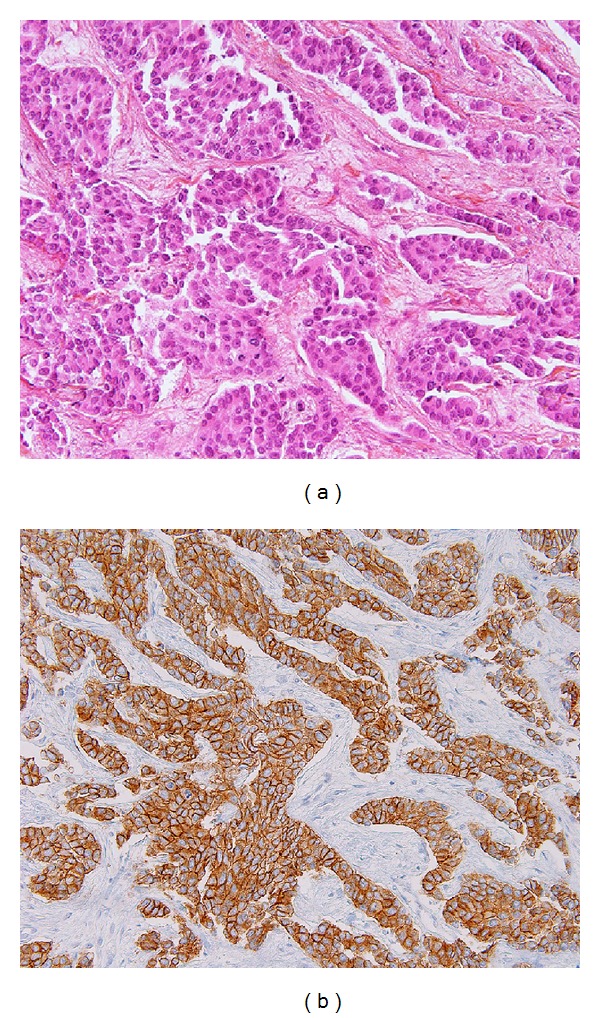
Breast tumor. (a) Biopsy specimen (staining with H&E, original magnification x200). (b) HER2 overexpression (3+) of tumor cells detected by immunohistochemistry (immunoperoxidase staining, primary monoclonal anti-HER2 antibody (clone CB11), original magnification x200).

**Figure 3:**
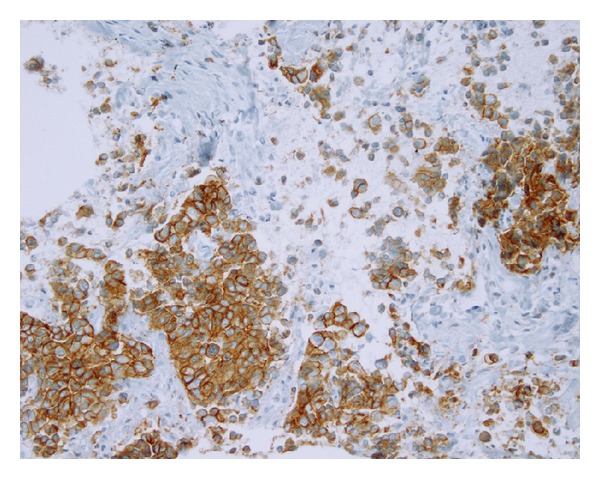
Tumor of the upper urinary tract: biopsy specimen with HER2 overexpression (3+) of tumor cells detected by immunohistochemistry (immunoperoxidase staining, primary monoclonal anti-HER2 antibody (clone CB11), original magnification x200).

A 60-year-old woman presented in December 2012 with macroscopic hematuria. CT-scan showed tumor of the right kidney extending to the peritoneum and the liver, second tumor in the urinary bladder, and pelvic and retroperitoneal enlarged lymph nodes. At the same time clinical examination and mammography (Figure 1) showed a rounded mass of the upper internal quadrant of the right breast. No other metastases were found. The patient underwent biopsy of all three lesions—breast, upper urinary tract, and bladder tumors. All samples showed poorly differentiated carcinoma with positive expression of CK7 and P63, 3+ HER2 staining (Figures 2 and 3), and no expression of estrogen or progesterone receptors. On the breast biopsy, no intracanalar tumor was present. The pathological diagnosis suggested an HER2-overexpressing bifocal UC with metastases to the retroperitoneal lymph nodes, the liver, and the right breast. Identity of the breast and urothelial tumors was further confirmed by molecular biology; similarly p.K120M mutation of P53 (c.359A>T, nonfunctional missense substitution, located in the DNA-binding domain) was found in both samples. HER2 overexpression was found to be associated with polysomy of chromosome 17, with no HER2 gene amplification. In both tumors HER2 mRNA expression levels were high. No FGFR3 mutations were found on the bladder tumor. The patient underwent chemotherapy with the intensified MVAC (methotrexate, vinblastine, doxorubicin, and cisplatin) regimen. A CT-scan performed after the six cycles showed partial regression of the upper urothelial tract lesion. Similarly the breast metastasis shrank from 15 mm to 7 mm. However 8 weeks after the final cycle the patient presented with right hemiparesis, leading to the diagnosis of brain metastases. Despite whole-brain irradiation and 2 additional lines of chemotherapy (gemcitabine and paclitaxel/trastuzumab), the patient died of progressive disease 12 months after initial diagnosis.

## 3. Discussion

When discovering a breast lesion upon diagnosis or during treatment of another malignancy, the diagnosis of breast metastasis should be considered. However the frequency of primary breast cancer makes it necessary to obtain pathological confirmation of the metastatic nature of such a lesion. Direct comparison of biopsy samples between the primary cancer and the supposedly metastatic breast lesion is essential, and their similarity is a strong argument in favor of the diagnosis of breast metastasis. If pathology and immunochemistry cannot rule out primary breast cancer and fully ensure identity of primary tumor and breast metastasis, as in our particular case, one should resort to molecular biology. The p.K120M mutation is a very rare P53 mutation. It was described exclusively in urinary tract, brain, and liver [6–9] and never described in breast carcinoma (IARC P53 mutation database). The presence of this specific mutation, in both samples (breast and urothelial tumors), outlined the probable urothelial nature of the tumor.

The exact prevalence of HER2 overexpression in UC is unclear, with studies reporting values ranging from 9% to 81% [10]. Using the same methods and criteria as in breast cancer a recent study of 1005 primary UC samples found HER2 overexpression in 9.2% of tumor samples and HER2 gene amplification rate by FISH in 5.1%, with a complete concordance between 3+ protein expression level and gene amplification [11]. In our case a 3+ HER2 overexpression was associated with polysomy of chromosome 17. HER2 overexpression in our particular case was paradoxically a confusing factor when asserting the urothelial nature of the tumor—because of the higher frequency of HER2 overexpression in breast cancer—and a mean to further ensure the identity of primary UC and breast metastasis thanks to the concordance between the immunochemical and molecular features of both lesions.

Although less rare than breast metastases, CNS metastases in patients with UC remain unusual, with incidence rates as low as 1.7% in historical autopsy series. In a review of the literature published in 2012 Sarmiento et al. counted 290 reported cases of UC with CNS metastases, 231 of them involving brain parenchyma [12]. To the best of our knowledge ours is the first case in which breast and brain metastases from UC were encountered in the same patient.
